# Range of Movement and Patient-Reported Outcomes in Shoulder Arthroplasty in the Elderly: A Comparison of Anatomical Versus Reverse Shoulder Replacements

**DOI:** 10.7759/cureus.24657

**Published:** 2022-05-02

**Authors:** Gavin Schaller, Rory Cuthbert, Arjun Puvanendran, Matt Ravenscroft, Dilraj Sandher, Barnes Morgan, Daoud Makki

**Affiliations:** 1 Trauma and Orthopaedics, West Hertfordshire Hospitals NHS Trust, London, GBR; 2 Trauma and Orthopaedics, Addenbrooke’s Hospital, Cambridge University Hospitals NHS Foundation Trust, London, GBR; 3 Trauma and Orthopaedics, Stepping Hill Hospital - Stockport, Manchester, GBR

**Keywords:** shoulder/elbow arthroplasty, elderly population, range of movement, shoulder injuries, reverse shoulder arthroplasty

## Abstract

Introduction

Shoulder arthroplasty is in increasing demand in the elderly given the rising prevalence of glenohumeral osteoarthritis (OA) across the population. There is a dearth of evidence in the current literature comparing anatomical total shoulder arthroplasty and reverse total shoulder arthroplasty (RTSA) in the elderly population. This study compares patient-reported outcome measures and range of movement (ROM) between anatomical and reverse total shoulder replacements in matched patient cohorts over the age of 70 years.

Materials and methods

This retrospective cohort study compares range of movement and patient-reported outcomes between anatomical total shoulder arthroplasty and reverse total shoulder arthroplasty in matched cohorts over the age of 70 years at a single institution between 2011 and 2015. Overall, 75 patients were recruited, including 44 patients with anatomical total shoulder arthroplasty and 31 patients with reverse total shoulder arthroplasty. Cohorts were matched according to age, preoperative Oxford Shoulder Score (OSS), and preoperative range of movement. The minimum clinical follow-up was one year.

Results

At one year, range of movement metrics in flexion, abduction, and external rotation all demonstrated greater improvements in the anatomical total shoulder arthroplasty cohort in comparison to the reverse total shoulder arthroplasty cohort (p<0.0001). No statistical difference in Oxford Shoulder Score was evident between cohorts.

Conclusion

Anatomical total shoulder arthroplasty demonstrates a significant improvement in range of movement in comparison to reverse total shoulder arthroplasty in matched elderly patient cohorts. However, there was no statistical difference in functional assessment scores between reverse shoulder replacement and anatomical shoulder replacement.

## Introduction

Shoulder arthroplasty in the form of a total shoulder arthroplasty (TSA) is most commonly indicated in patients with severe primary glenohumeral osteoarthritis (OA) [1]. TSA requires a functioning rotator cuff to prevent the migration of the humeral component. In cuff arthropathy or cuff-deficient OA, when the rotator cuff is inadequately functioning, reverse total shoulder arthroplasty (RTSA) can be performed, which moves the center of rotation medially and inferiorly, improving the stability of the joint.

The prevalence of both TSA and RTSA continues to rise, with a 5.6-fold increase over a 19-year period between 1998 and 2017 in the United Kingdom [2]. In the context of an increasing population life expectancy, the number of elderly patients with OA requiring shoulder replacement will continue to climb [3]. Arthroplasty surgery has been shown to deliver improved patient outcomes by reducing persistent shoulder pain and improving function, activities of daily living (ADLs), and range of movement (ROM) [4]. There are numerous scoring systems available to assess shoulder satisfaction and function, including the Oxford Shoulder Score (OSS). The OSS is a validated 12-item questionnaire to evaluate outcomes in which patients report on their pain and impact on daily function [5].

TSA has been shown to be effective in the elderly population for improving pain and range of movement in patients with severe OA [6]. RTSA was initially introduced into Europe in 1987 and was primarily indicated for rotator cuff arthropathy [7]. More recently, RTSA has broadened its indications to include acute shoulder fractures, revision surgery, and inflammatory arthropathies [8].

There is currently a lack of significant literature comparing TSA and RTSA in the elderly population. The aim of this study was to assess and compare postoperative functional and ROM outcomes between a matched patient cohort aged over 70 years who had undergone TSA and RTSA.

## Materials and methods

This study was undertaken at a single orthopedic general hospital between January 2011 and July 2015 by orthopedic consultants subspecializing in shoulder surgery. Local ethical approval was obtained.

All patients aged over 70 years who had undergone primary shoulder arthroplasty between January 2011 and July 2015 were included. The exclusion criteria were as follows: all patients who had undergone revision shoulder arthroplasty and all patients undergoing shoulder arthroplasty for fracture or inflammatory arthropathy.

All patients who had undergone TSA underwent preoperative magnetic resonance imaging (MRI) of the shoulder. The integrity of the rotator cuff was evaluated on MRI and staged in accordance with Goutallier’s classification of fatty infiltration of the rotator cuff musculature [9]. Only patients with less than or equal to 50% fatty muscle atrophy (Goutallier stage 3) and a confirmed intact rotator cuff were deemed suitable for TSA. Patients who had undergone RTSA underwent preoperative radiographs demonstrating rotator cuff arthropathy or had MRI/ultrasound scans confirming cuff deficiency in the presence of glenohumeral degenerative changes. Table 1 demonstrates preoperative Goutallier grading and intraoperative cuff findings for all cases of TSA (classified as normal, partial articular supraspinatus tendon avulsion (PASTA) less than 50%, and PASTA 50%) (Table 1).

**Table 1:** Preoperative Goutallier grading and intraoperative cuff findings for TSA

Preoperative Goutallier grading	Number of TSA performed	Intraoperative rotator cuff findings		
		Normal cuff	PASTA lesion (<50%)	PASTA lesion (50%)
1	14 (32%)	10	3	1
2	17 (39%)	8	4	5
3	13 (29%)	10	2	1

Each patient cohort was followed up after surgery for a minimum of a one-year period with OSS recorded preoperatively and at three months, six months, and one year postoperatively. Active ROM was measured using a goniometer by two independent extended-scope shoulder practitioners preoperatively and at one-year follow-up.

Statistical analysis

The TSA and RTSA cohorts were matched according to age, preoperative OSS, and preoperative ROM. Postoperative OSS and ROM were compared between the TSA and RTSA cohorts using the unpaired two-sample t-test. Within each cohort, improvements in OSS and ROM were compared to preoperative OSS and ROM using the paired one-sample t-test.

Surgical technique

All TSA and RTSA operations were conducted under general anesthesia and regional block. A deltopectoral approach was employed. The implant used in all patients was the Zimmer Biomet Comprehensive Shoulder Replacement System [10]. A standardized postoperative rehabilitation regime was implemented for all patients, with a broad arm sling used for the first week. Subsequently, pendular exercises were commenced until the end of the second week, followed by an active range of movement under the direction of the physiotherapy team.

## Results

Shoulder arthroplasty surgery was performed on 75 patients. Forty-four patients underwent TSA, while 31 patients underwent RTSA. There was no loss to follow-up. The mean age of patients was 74.1 years (range: 70-88 years) in the TSA cohort and 76.3 years (range: 70-90 years) in the RTSA cohort.

The mean preoperative OSS in the TSA cohort was 13. At one-year follow-up, this had risen to 41. The mean preoperative OSS in the RTSA cohort was 16. At one-year follow-up, this had risen to 39 (Table 2). The OSS at each stage of follow-up demonstrated no statistical difference between cohorts.

**Table 2:** Mean OSS in the TSA and RTSA cohorts

	Preoperative OSS	Three-month OSS	Six-month OSS	One-year OSS
TSA cohort (n=44)	13	31	35	41
RTSA cohort (n=31)	16	32	35	39

At one-year follow-up, the mean ROM in the TSA cohort was flexion to 128 degrees, abduction to 122 degrees, and external rotation to 40 degrees (Table 2). The unpaired two-sample t-test demonstrated a statistically significant improvement in flexion, abduction, and external rotation in the TSA cohort compared to the RTSA cohort (p<0.0001).

**Table 3:** Mean ROM at one year in the TSA and RTSA cohorts

	Flexion	Abduction	External rotation
TSA cohort (n=44)	128	122	40
RTSA cohort (n=31)	112	103	26

Postoperative complications were identified within the one-year follow-up period in the TSA cohort: two patients experienced dislocations due to subscapularis failure with one patient undergoing revision to RTSA. Further, one patient suffered transient axillary and musculocutaneous nerve neuropraxia, which resolved prior to the first postoperative follow-up appointment, and one patient developed a superficial wound infection, which also resolved. There were no complications in the RTSA cohort and no postoperative mortalities within the follow-up period.

## Discussion

The growing demand for shoulder replacement surgery in the elderly is evident secondary to the growing population life expectancy. This study compared patient-reported outcome measures and range of movement between TSA and RTSA in matched patient cohorts over the age of 70 years.

In both cohorts, OSS improvement was evident at each stage of follow-up. This was not statistically significant between cohorts, which may reflect the fact that OSS is an assessment of pain and impact on ADL. Therefore, reduced ROM may not necessarily have been perceived as a functional deficit in an elderly cohort likely to have lower demands for ROM compared to a younger population.

ROM was improved in both patient cohorts with a statistically significant improvement in flexion, abduction, and external rotation evident in the TSA cohort compared to the RTSA cohort. Kiet et al. demonstrated that anatomical shoulder replacements can improve ROM metrics such as external rotation due to the comparatively less medial center of rotation [11]. Further, Kiet et al. demonstrated that complication rates, need for revision, and patient-reported outcomes were similar between the two cohorts at two years of follow-up. Similarly, Flurin et al. also demonstrated similar clinical outcome metrics between TSA and RTSA [12].

Current trends suggest that the incidence of RTSA is increasing in comparison to TSA [13]. This is likely to be due to clinicians maintaining a lower threshold to proceed to RTSA when the quality of the rotator cuff is questionable as TSA carries the increased risk of revision surgery following cuff failure. Our study demonstrates that despite a loss in ROM, patients report comparable functional outcomes in RTSA compared to TSA. Further, the complication rate was higher in the TSA cohort. Therefore, in cases where the patency of the rotator cuff is questionable, consideration of RTSA in the elderly is a valid surgical option.

There are limitations to this study. Firstly, this was a retrospective cohort study based on a small sample size in each cohort. Further, patients were only followed up for one year. Future research should seek to establish long-term outcomes in TSA and RTSA patient cohorts. Despite these limitations, this study makes an important contribution to the literature as it remains one of the few studies to present a matched patient cohort comparing TSA and RTSA in the elderly.

## Conclusions

Anatomical total shoulder arthroplasty demonstrates a significant improvement in range of movement compared to reverse total shoulder arthroplasty in matched elderly patient cohorts. However, there is no statistical difference in functional assessment scores between reverse shoulder replacement and anatomical shoulder replacement. Therefore, if there is concern over the quality of the rotator cuff either preoperatively or intraoperatively, RTSA should be considered a viable option in the elderly population, which offers similar functional outcomes.

## References

[1] Mattei L, Mortera S, Arrigoni C, Castoldi F (2015). Anatomic shoulder arthroplasty: an update on indications, technique, results and complication rates. Joints.

[2] Craig RS, Lane JC, Carr AJ, Furniss D, Collins GS, Rees JL (2019). Serious adverse events and lifetime risk of reoperation after elective shoulder replacement: population based cohort study using hospital episode statistics for England. BMJ.

[3] Ricchetti ET, Abboud JA, Kuntz AF, Ramsey ML, Glaser DL, Williams GR Jr (2011). Total shoulder arthroplasty in older patients: increased perioperative morbidity?. Clin Orthop Relat Res.

[4] Simovitch RW, Friedman RJ, Cheung EV, Flurin PH, Wright T, Zuckerman JD, Roche C (2017). Rate of improvement in clinical outcomes with anatomic and reverse total shoulder arthroplasty. J Bone Joint Surg Am.

[5] Angst F, Schwyzer HK, Aeschlimann A, Simmen BR, Goldhahn J (2011). Measures of adult shoulder function: Disabilities of the Arm, Shoulder, and Hand Questionnaire (DASH) and its short version (QuickDASH), Shoulder Pain and Disability Index (SPADI), American Shoulder and Elbow Surgeons (ASES) Society standardized shoulder assessment form, Constant (Murley) Score (CS), Simple Shoulder Test (SST), Oxford Shoulder Score (OSS), Shoulder Disability Questionnaire (SDQ), and Western Ontario Shoulder Instability Index (WOSI). Arthritis Care Res (Hoboken).

[6] Foruria AM, Sperling JW, Ankem HK, Oh LS, Cofield RH (2010). Total shoulder replacement for osteoarthritis in patients 80 years of age and older. J Bone Joint Surg Br.

[7] Khan WS, Longo UG, Ahrens PM, Denaro V, Maffulli N (2011). A systematic review of the reverse shoulder replacement in rotator cuff arthropathy, rotator cuff tears, and rheumatoid arthritis. Sports Med Arthrosc Rev.

[8] Drake GN, O'Connor DP, Edwards TB (2010). Indications for reverse total shoulder arthroplasty in rotator cuff disease. Clin Orthop Relat Res.

[9] Somerson JS, Hsu JE, Gorbaty JD, Gee AO (2016). Classifications in brief: Goutallier classification of fatty infiltration of the rotator cuff musculature. Clin Orthop Relat Res.

[10] Zimmer Biomet (2019). Zimmer Biomet: Shoulder replacement products. http://www.zimmerbiomet.com/medical-professionals/shoulder.html.

[11] Kiet TK, Feeley BT, Naimark M, Gajiu T, Hall SL, Chung TT, Ma CB (2015). Outcomes after shoulder replacement: comparison between reverse and anatomic total shoulder arthroplasty. J Shoulder Elbow Surg.

[12] Flurin PH, Roche CP, Wright TW, Marczuk Y, Zuckerman JD (2015). A comparison and correlation of clinical outcome metrics in anatomic and reverse total shoulder arthroplasty. Bull Hosp Jt Dis (2013).

[13] (2019). National Joint Registry: Shoulders - Primary procedures - Activity. https://reports.njrcentre.org.uk/shoulders-primary-procedures-activity.

